# Core Site-Moiety Maps Reveal Inhibitors and Binding Mechanisms of Orthologous Proteins by Screening Compound Libraries

**DOI:** 10.1371/journal.pone.0032142

**Published:** 2012-02-29

**Authors:** Kai-Cheng Hsu, Wen-Chi Cheng, Yen-Fu Chen, Hung-Jung Wang, Ling-Ting Li, Wen-Ching Wang, Jinn-Moon Yang

**Affiliations:** 1 Institute of Bioinformatics and Systems Biology, National Chiao Tung University, Hsinchu, Taiwan; 2 Department of Life Sciences, Institute of Molecular and Cellular Biology, National Tsing Hua University, Hsinchu, Taiwan; 3 Department of Biological Science and Technology, National Chiao Tung University, Hsinchu, Taiwan; Università di Napoli Federico II, Italy

## Abstract

Members of protein families often share conserved structural subsites for interaction with chemically similar moieties despite low sequence identity. We propose a core site-moiety map of multiple proteins (called CoreSiMMap) to discover inhibitors and mechanisms by profiling subsite-moiety interactions of immense screening compounds. The consensus anchor, the subsite-moiety interactions with statistical significance, of a CoreSiMMap can be regarded as a “hot spot” that represents the conserved binding environments involved in biological functions. Here, we derive the CoreSiMMap with six consensus anchors and identify six inhibitors (IC_50_<8.0 *µ*M) of shikimate kinases (SKs) of *Mycobacterium tuberculosis* and *Helicobacter pylori* from the NCI database (236,962 compounds). Studies of site-directed mutagenesis and analogues reveal that these conserved interacting residues and moieties contribute to pocket-moiety interaction spots and biological functions. These results reveal that our multi-target screening strategy and the CoreSiMMap can increase the accuracy of screening in the identification of novel inhibitors and subsite-moiety environments for elucidating the binding mechanisms of targets.

## Introduction

The expanding number of protein structures and advances in bioinformatics tools have offered an exciting opportunity for structure-based virtual screening in drug discovery [1]. Although there are some successful agents in the antibiotic development, few agents act at novel molecular binding sites to target multiple antibiotic–resistant pathogenic bacteria [2], [3]. However, screening tools are often designed for one-target paradigm and the scoring methods are highly target-dependent and energy-based. As a result, they cannot consistently and persuasively identify true leads, leading to a low success rate [4]–[6].

Orthologous proteins often perform similar functions, despite low sequence identity. Importantly, they frequently share conserved binding environments for interacting with partners. These proteins and their interacting partners (inhibitors or substrates) can be regarded as a pharmacophore family, which is a group of protein-compound complexes that share similar physical-chemical features and interaction patterns between the proteins and their partners. Such a family is analogous to a protein sequence family [7], [8] and a protein structure family [9]. However, the establishment of pharmacophores often requires a set of known active ligands that were acquired experimentally [10]–[12]. Developing an efficient method for identifying new adaptive inhibitors against multiple targets from public compound libraries is therefore becoming an important task [13]–[15].

To address the above issues, we propose a core site-moiety map to discover inhibitors and mechanisms of multiple targets from large-scale docked compounds. The consensus anchors, which are subsite-moiety interactions with statistical significance in site-moiety maps of these proteins, represent the conserved binding environments that are involved in biological functions. The new method (called CoreSiMMap-based screening method) was heavily modified and improved from that “SiMMap” in our earlier work [16], which constructed a site-moiety map comprising of anchors from a target protein and thousands of docked compounds. An anchor contains three crucial elements, which are conserved interacting residues that constitute a binding pocket (part of the binding site), the preference of moieties, and a pocket-moiety interaction type.

The major enhancements of the CoreSiMMap for multi-target inhibitors from SiMMap are as follows: 1) we developed the robust theoretical model for the SiMMap; 2) the CoreSiMMap is designed for multiple target proteins by modifying the SiMMap on a single target protein; 3) we added an anchor alignment method to identify core binding environments (anchors) among multiple targets to reveal binding mechanisms; 4) we added a rank-based consensus score (RCS) of multiple targets to improve the enrichment of true positives. Based on these enhancements and modifications, the CoreSiMMap-based screening method is useful to infer core pharmacophores both to identify adaptive inhibitors of multiple targets and to improve screening accuracy.

Here, we have applied the CoreSiMMap strategy to discover core pharmacophores and adaptive inhibitors of shikimate kinase (SK) of *Mycobacterium tuberculosis* and *Helicobacter pylori* (MtSK and HpSK) by screening large compound libraries. Mt causes tuberculosis and killed 1.7 million people in 2006 [17]. Therefore, it is becoming a major public health threat[18]. We first derived core site-moiety maps that often represent the conserved binding environment elements or “hot spots” among orthologous targets based on virtual screening. In using core site-moiety maps, six potent adaptive inhibitors of MtSK and HpSK with low IC_50_ values (<8.0 *µ*M) were identified. Site-directed mutagenesis revealed that the core pharmacophores often contribute to specific pocket-moiety interaction anchors. These results reveal the CoreSiMMap is useful to identify adaptive SK inhibitors and provide insight into the binding mechanisms of compounds.

## Results

### Overview of CoreSiMMap

**Figure 1:**
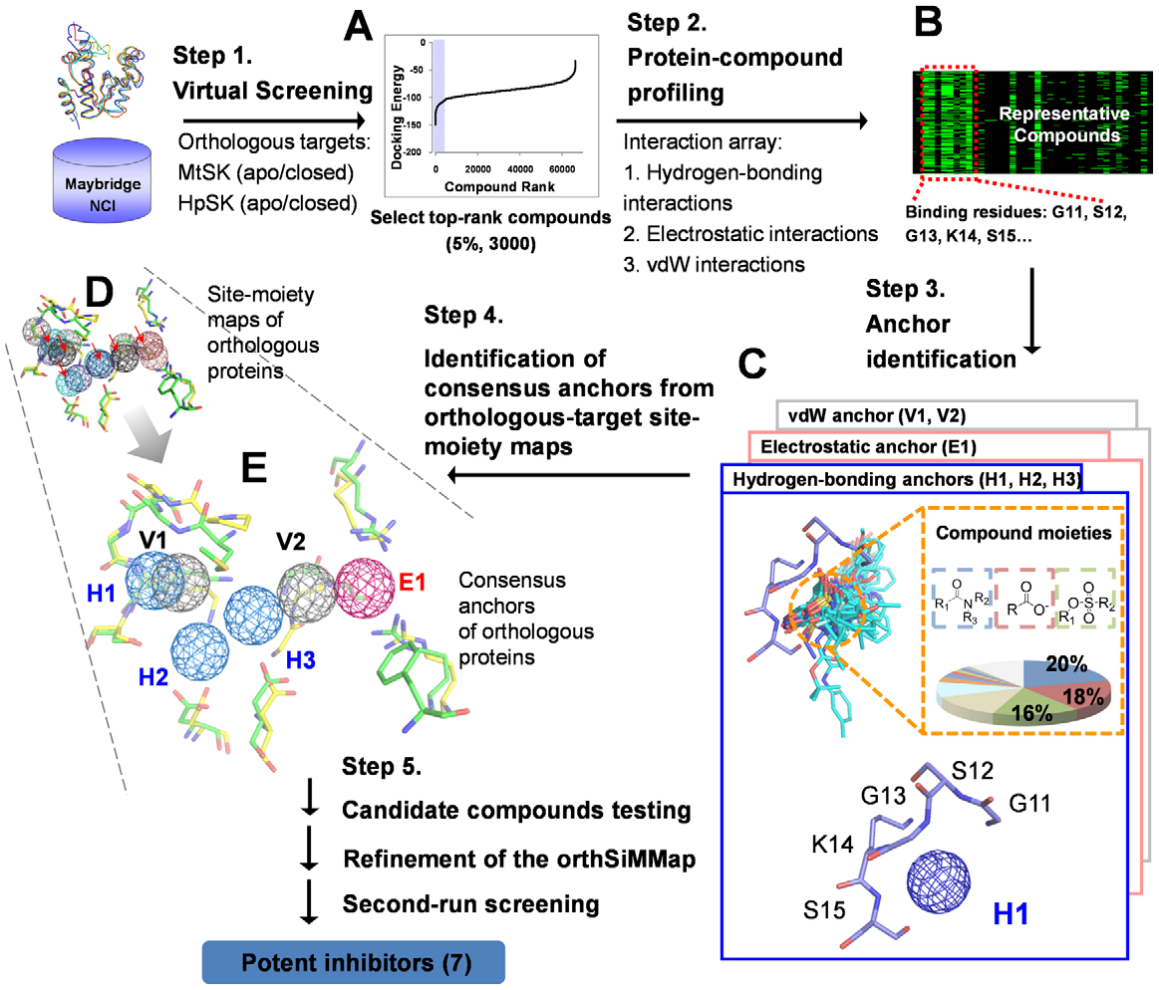
Framework of CoreSiMMap-based screening method. In Step 1, GEMDOCK was used to generate docked poses for HpSK and MtSK by screening compound libraries (Maybridge and NCI). For each target (HpSK or MtSK), the protein-compound interacting profile was derived by the top 2% (∼6,000) of compounds ranked by docking energy. In Step 3, conserved interactions of the target protein and chemical moieties of ligands are identified to deduce the anchors of HpSK and MtSK. The CoreSiMMap is constructed based on the features that are conserved between orthologous target site-moiety maps, which will be used to select candidate compounds for the enzymatic assay. Finally, the model is refined based on the bioassay of candidate compounds.

**Figure 2:**
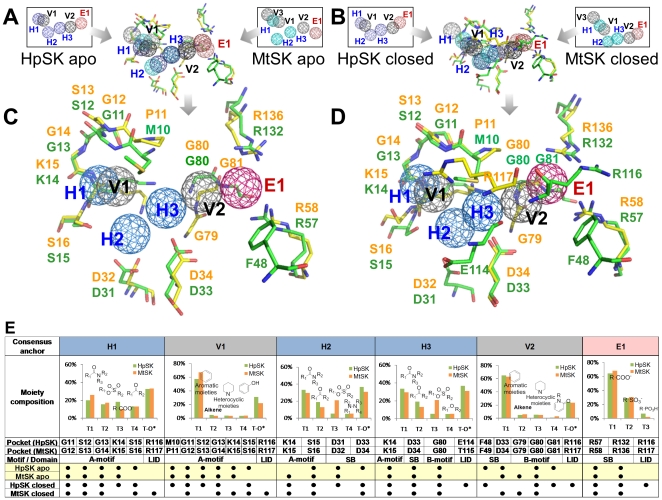
Shikimate kinase CoreSiMMaps. (A) Superimposed apo-form anchors of HpSK and MtSK. (B) Superimposed closed-form anchors of HpSK and MtSK. (C) The apo-form CoreSiMMap and (D) the closed-form CoreSiMMap include six consensus anchors derived from consensus anchors (A) and (B), respectively. Each consensus anchor shares conserved residues between HpSK and MtSK and has the same interaction type of binding environment. (E) Features of the six consensus anchors of the apo-form CoreSiMMap. Each of the T groups (T1–T4) represents a given chemical moiety and T-O* indicates other chemical groups. H1, V1, and H2 are situated at the ATP-binding site, while H3, V2, and E1 are at the shikimate-binding site. Each consensus anchor includes conserved interacting residues (•) and the major chemical moieties of the compound candidates.

A CoreSiMMap is the consensus site-moiety maps, which consist of several consensus anchors derived from multiple targets, to represent essential features that are involved in the common biological functions of these targets (Figs. 1 and 2). The following criteria are considered for a CoreSiMMap: (1) the binding sites of the screening targets share conserved physical-chemical features; (2) pocket-moiety interaction profiles of these targets and well-docked compounds are similar; and (3) the site-moiety maps of these targets share comparable anchors (pharmacophores) with respect to their sites and crucial protein-ligand interactions.

Figure 1 presents the major steps of the CoreSiMMap-based method for orthologous targets (HpSK and MtSK). For each target, we used the top 2% (∼6000) of compounds obtained by screening compound libraries to analyze target-compound interaction profiles (Fig. 1B) to establish the site-moiety maps and “pharmacophore spots (anchors) (Fig. 1C)” with statistically significant Z-scores. The superimposed pharmacophore models with anchors among orthologous proteins revealed overlapping regions, which are regarded as “hot spots”. A set of hot spots from orthologous targets therefore form a core site-moiety map that represents the conserved binding elements (Fig. 1E). After enzyme inhibition assay, active and inactive compounds were used to refine the CoreSiMMap model and core anchors.

### CoreSiMMaps of HpSK and MtSK

The CoreSiMMap that has comparable spots or hot spots is useful in providing biological insights and guiding the process of drug discovery including hit search and lead optimization. Here, we utilized the CoreSiMMap strategy to identify adaptive inhibitors of MtSK and HpSK. The SK is the fifth enzyme in the shikimate pathway and converts shikimate to shikimate 3-phosphate[19]. The SK is an attractive target for the development of new antimicrobial agents, herbicides, and antiparasitic agents since it is essential to bacteria, fungi, and plants, but not animals [20]. Because there are distinct binding pockets with or without ligands for shikimate kinase, we sought to establish apo-form and closed-form CoreSiMMaps of SKs (Fig. 2). We first generated the apo-form SiMMaps of HpSK (6 anchors) and MtSK (7 anchors), allowing us to derive the CoreSiMMap with 6 consensus anchors (Figs. 2A and 2C). In parallel, the closed-form CoreSiMMap with 6 consensus anchors was also derived (Figs. 2B and 2D).

The apo-form and closed-form CoreSiMMaps have six comparable anchors: E1, H1–H3, V1, and V2 (Figs. 2C and 2D). H1, H2, and V1 sit at the ATP site, while H3, V2, and E1 are situated at the shikimate site (Figs. S1 and S2). The protein-ligand relationship was analyzed for each hot spot; a set of chemically related entities that contribute to intermolecular interactions were then identified (Figs. 2E, S1, and S2). Our results support the notion that a hot spot has a conserved binding environment with a specific chemical-physical property, which can be used as a guide in further lead optimization. The compounds moieties, anchors, SiMMaps and CoreSiMMaps are available at http://simmap.life.nctu.edu.tw/orthsimmap/.

Of the six consensus anchors (Fig. 2E), E1 is a negatively charged pocket that interacts with R57 (R58 in MtSK), R116 (R117 in MtSK), and R132 in HpSK (R136 in MtSK); these arginines are highly conserved in SKs and are critical for binding to shikimate [21] (Fig. 2, S1, and S2). The chemical entities on E1 consisted of carboxyl, sulfonate, and phosphate groups. H1 is enclosed with a tight turn (Walker A motif) that binds the β-phosphate of ATP [21]. The identified moieties were carboxylic amide, sulfonate ester, carboxyl acid, and ketone (Fig. 2E). H2 is located between H1 and H3 and possesses a hydrogen-bonding environment from a Walker A motif (K14 and S15 in HpSK; K15 and S16 in MtSK) and a DT/SD motif (D31 and D33 in HpSK; D32 and D34 in MtSK). Amide, ketone, sulfonate ester, and azine-containing compounds fit into this pocket. H3 is situated above the central sheet in which two conserved residues (D33, and G80 in HpSK; D34, G80 in MtSK) contribute to H3. Amide, sulfonate ester, and ester groups were frequently identified.

V1, which is adjacent to H1, bears a vdW-binding environment and also contains residues from the Walker A motif. V2, which is near H3, is situated at the border between shikimate and the nucleotide binding regions. V1 and V2, allowing the interactions with large chemical groups, prefer aromatic groups. Analysis of the closed-form SiMMaps indicates that E114 and R116 (T115 and R117 in MtSK) in LID are conserved interacting residues (Fig. 2E). Analysis of the closed-form model shows that the four hot spots (E1, H1, H2, and V1) occupy a similar region in the closed-form pharmacophores. Given the closure of the LID region, E1, H2, and V1, interact with a residue from LID [19], [22]. On the other hand, H3 and V2 were absent in the closed form, perhaps because of its tight binding pocket.

### Inhibitors and inhibition assay

**Figure 3:**
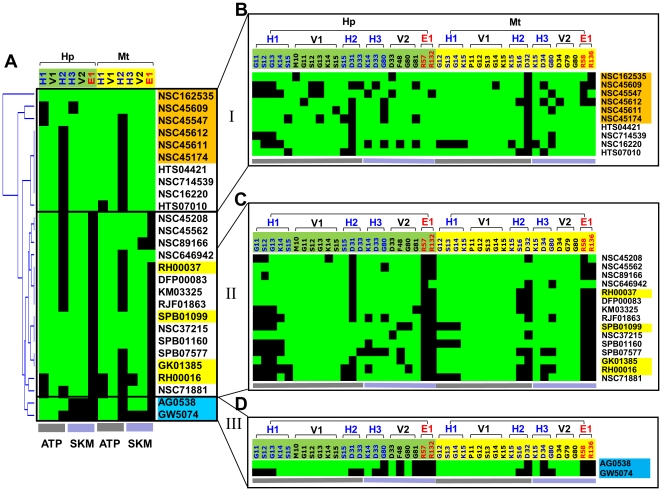
Interaction profiles between selected anchor residues and 27 tested compounds. (A) Anchor profile of tested compounds on shikimate kinases. (B) Group I: NCI compounds (orange). (C) Group II: Maybridge compounds (yellow). (D) Group III: kinase inhibitors (cyan). The NCI compounds consistently occupy anchors E1 and V2 at both ATP and shikimate sites. The NCI compounds except NSC45174 are competitive inhibitors with both ATP and shikimate. For the Maybridge compounds, none form electrostatic interactions with R57 and R132 on the consensus anchor E1. The two kinase compounds are located at the ATP site, which fact is consistent with the kinetic results that reveal that these compounds exhibited competitive inhibition with ATP and noncompetitive inhibition with shikimate.

**Figure 4:**
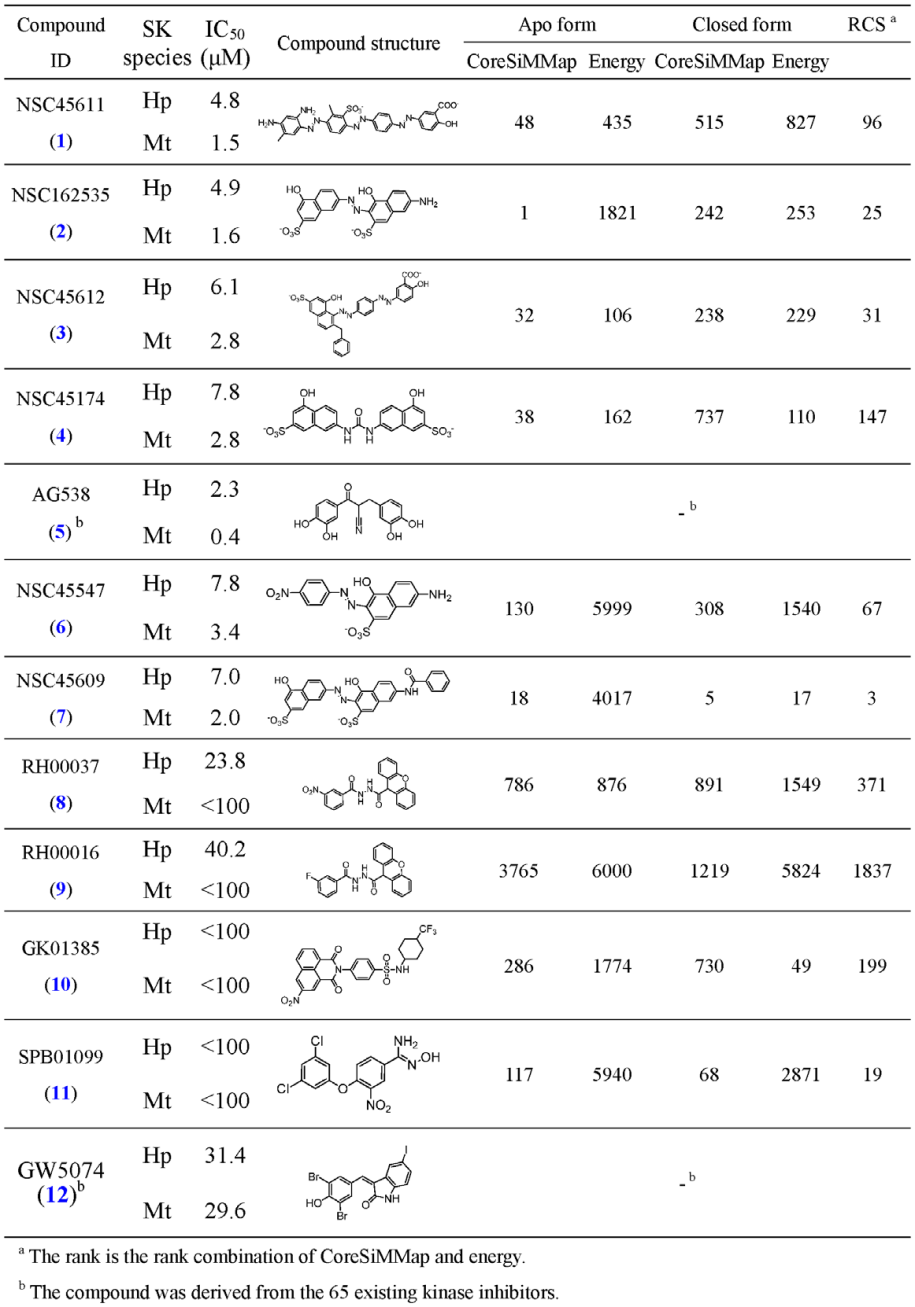
Ranks of active compounds using CoreSiMMap, energy-based, and combined scoring methods for apo and closed forms of HpSK and MtSK.

**Figure 5:**
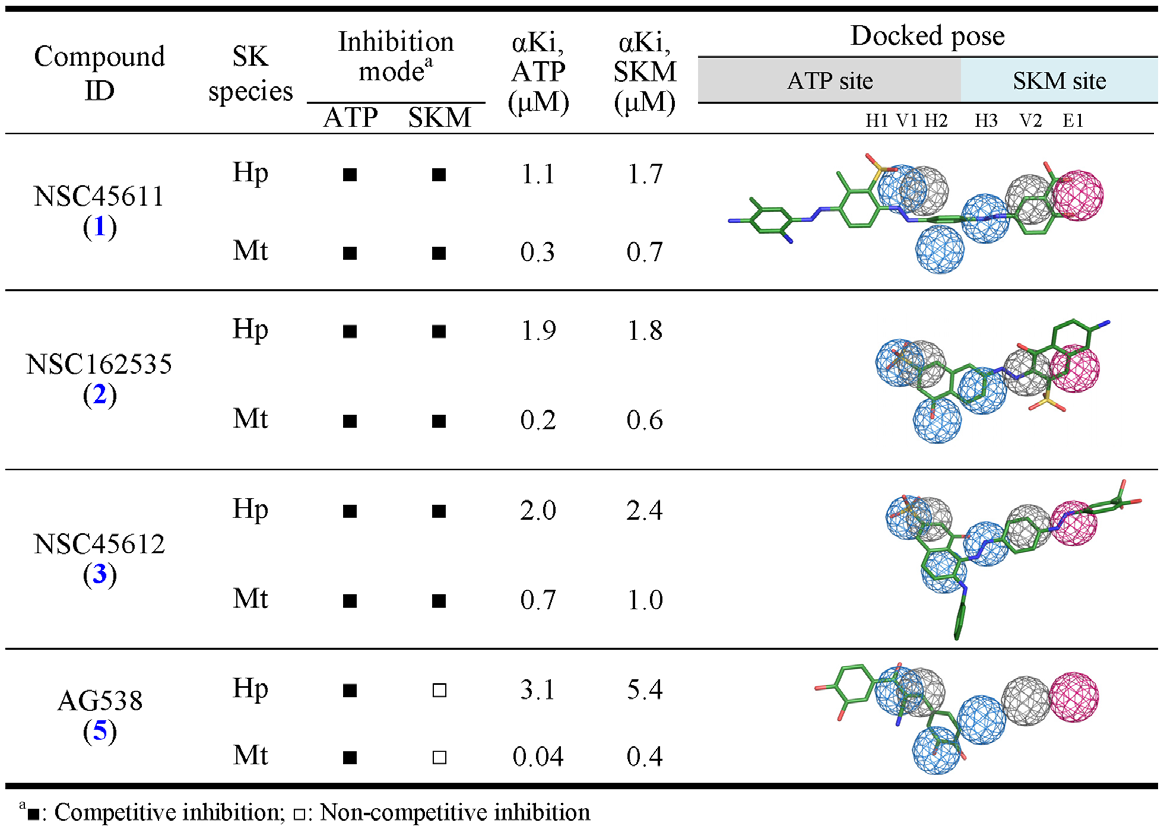
Properties of some potent inhibitors of HpSK and MtSK. Docked poses of NSC45611, NSC162535, and NSC45612 are located at the ATP site (H1, H2, and V1) and the shikimate site (H3, V2, and E1), and these inhibitors are competitive inhibitors of ATP and shikimate (SKM). AG538 is a competitive inhibitor of ATP, in agreement with its docked pose.

Following the analyses of the SiMMap and the compound-anchor-residue profiles (Fig. 3), we used the CoreSiMMap in post-screening analysis to rescore docked compounds by the rank-based consensus scoring (RCS [23]), which combines energy-based and anchor-based scoring (Fig. 4). Since a compound that simultaneously docks into apo and closed-form binding sites of HpSK and MtSK is regarded as a potentially useful hit, we selected common top-ranked compounds from the closed-form and apo-form CoreSiMMap analysis for subsequent bioassay. After compounds were ranked by using RCS in both the Maybridge and the NCI databases, 48 available compounds (either requested or purchased) were subjected to MtSK and HpSK inhibitory assays. Among those, 10 compounds had IC_50_ ≤100 µM for both HpSK and MtSK, of which six [NSC45611 (**1**), NSC162535 (**2**), NSC45612 (**3**), NSC45174 (**4**), NSC45547 (**6**), and NSC45609 (**7**)] had IC_50_≤10 µM. In parallel, 65 existing kinase inhibitors were tested to evaluate their inhibitory effectiveness against shikimate kinase. Of the two compounds [AG538 (**5**) and GW5074 (**12**)] that showed inhibitory effects, AG538 (**5**) had a low IC_50_ value. Enzymatic kinetic analysis revealed that NSC45611 (**1**), NSC162535 (**2**), NSC45612 (**3**) and AG538 (**5**) were competitive inhibitors of ATP, in agreement with the docked poses (Fig. 5). Of these, NSC45611 (**1**), NSC162535 (**2**) and NSC45612 (**3**) competed with shikimate and had low IC_50_ and α*K_i_* values, showing potent inhibition.

**Figure 6:**
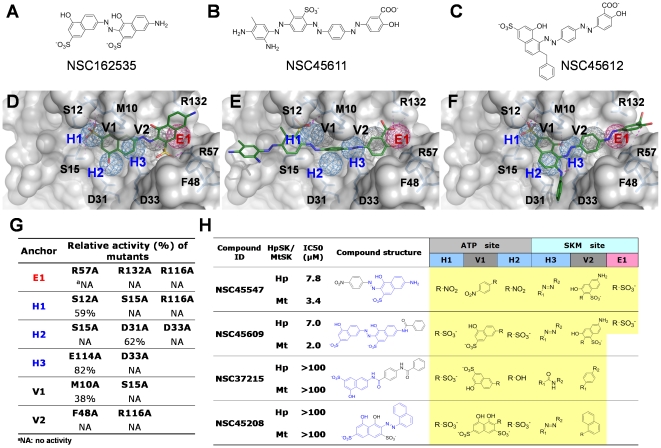
Characterization of shikimate kinase inhibitors by enzyme assay, CoreSiMMaps, site-mutagenesis studies, and analogues. (A–C) Structures of three inhibitors, NSC162535 (**2**), NSC45611 (**1**), and NSC45612 (**3**). (D–F) Relationships between anchors and docked mode of each inhibitor for HpSK. These compounds consistently include two negative charge moieties (SO_3_^−^ or CO_2_^−^) that form hydrogen bonds with conserved interacting residues of anchors E1 and H1. (G) Comparison of relative activities of HpSK mutants. The conserved interacting residues for each anchor were mutated. R57, R132, R116, and F48 located in the shikimate site were critical for the enzymatic functions. (H) Potency of NSC162535 analogues. The substitution moieties of analogues are indicated in black. Those that lack the E1 moiety greatly lost the inhibitory effects (IC_50_>100 µM).

We then further evaluated the binding and pharmacophore modes of these inhibitors. Figure 6 shows that these three compounds have lower IC_50_ (≤10 µM) and fit well into five hot spots (H1, V1, H3, V2, and E1). The compounds with IC_50_≥20 µM lack the negatively charged groups that are required to form electrostatic interactions with arginines (R57 and R136 in HpSK) on E1. On the other hand, kinase inhibitors AG538 (**5**) and GW5074 (**12**) did not occupy the shikimate site. Moieties with 1–3 rings were present at V1 and V2, yielding a number of vdW contacts. The binding groups of active inhibitors matched well with the identified moieties that were found from the consensus anchors. For example, the sulfonate groups of NSC162535 (**2**), NSC45611 (**1**), and NSC45612 (**3**) were found to occupy H1. The moieties of NSC162535 (**2**) (SO_3_^−^ group), NSC45611 (**1**) (CO_2_^−^ group), and NSC45612 (**3**) (CO_2_^−^ group) occupied E1.

### Consensus residues of CoreSiMMap and site-directed mutagenesis

A consensus anchor of orthologous targets that is identified from the conserved binding pockets has conserved interacting residues and a specific physico-chemical property, which often engage in specific enzymatic functions. We sought to investigate the roles of identified consensus anchor residues of the CoreSiMMaps in catalysis (Fig. 2). Each of the selected residues was replaced with alanine and expressed in *Escherichia coli*. After purification using affinity chromatography, all mutants migrated to a major band of an apparent molecular mass of ∼18 kDa in SDS-PAGE, as expected. We first investigated mutants of E1 residues (R57, R116, and R132) that contact with shikimate [19]. Enzymatic analysis revealed that these arginines had extremely low activity (Fig. 6G), suggesting their importance in catalysis. Indeed, R117 of MtSK, which corresponds to R116 of HpSK, has thus been suggested as a primary candidate to stabilize the transition-state intermediate [24].

For the H3 (D33) and V2 (F48) residues, D33A completely lost the enzymatic activity while F48A exhibited hardly any detectable activity (1%, Fig. 6G). D33 and F48 are in direct contact with shikimate. More importantly, D33 forms a hydrogen bond with the 3-OH group of shikimate, which may increase the nucleophilicity of the O atom or accept the proton from the 3-OH group of shikimate, facilitating catalysis. E114A, a LID residue whose side chain faces the solvent, retained 82% relative activity. On the other hand, the F48 side chain contacts with those from several residues nearby (V44, E53, F56, R57 and P117), potentially forming a stable platform that interacts with the ligand for a subsequent catalytic reaction.

We then evaluated residues from H1, H2, and V1 at the nucleotide site. H1 residues are primarily from the Walker A motif (P loop; residues 11–16, GSGKSS) that surrounds the phosphate groups of the nucleotides. Of the two alanine mutants (S12A and S15A), S12A retained 59% of the relative activity, while S15A had extremely low activity (1%). The S15 side chain resides near the β-phosphate of ADP. Furthermore, the adjacent lysine (K14) corresponding to K15 of MtSK has been identified as a critical catalytic residue in MtSK since its side chain points toward the γ-phosphate [24]. The other H2 mutant, D31A, retained 62% of the relative activity, possibly because of its remoteness from the phosphate group. For V1 that is just next to H1, several H1 residues are also shared by V1. Enzymatic analysis indicated that M10A retained 38% relative activity. These site-directed mutagenesis studies revealed that the critical conserved interacting residues of a pocket-moiety interaction spot participated in biological functions.

### Analogues assay and CoreSiMMap

To verify the moiety preferences of consensus anchors, we identified four analogues of NSC162535 (**2**) [NSC45547 (**6**), NSC45609 (**7**), NSC37215, and NSC45208] for inhibitory assays (Fig. 6H). NSC45547 (**6**) and NSC45609 (**7**) that occupy E1 (SO_3_^−^ group) and H1 (SO_3_^−^ and NO_2_ groups) retained good IC_50_ values (7.8 and 7.0 µM for HpSK; 3.4 and 2.0 µM for MtSK). Conversely, NSC37215 and NSC45208, which cannot anchor at E1, lost the inhibitory.

To evaluate the significance of the pocket-moiety interaction preferences of consensus anchors in the CoreSiMMaps, we performed clustering analysis on 27 inhibitory assay compounds (Fig. 3). These compounds can be roughly clustered into three groups. The potent inhibitors of Group I [NSC162535 (**2**), NSC45609 (**7**), NSC45547 (**6**), NSC45174 (**4**), NSC45611 (**1**) and NSC45612 (**3**)] match more than 5 consensus anchors (Fig. 3B). Each of the Group II compounds [RH00037 (**8**), RH00016 (**9**), GK01385 (**10**), and SPB01099 (**11**)] matches four of six anchors; Group III comprises kinase inhibitors [AG538 (**5**) and GW5074 (**12**)] and these compounds match anchors at the ATP site. The inactive compounds often match few consensus anchors in the HpSK/MtSK (usually 4), in particular, E1 is the least seen anchor. While the inhibitors of Group I and II match anchors at the ATP site and the shikimate site, kinetic assay indicates competitive inhibitions for ATP and shikimate acid (Fig. 5). The kinase inhibitors of Group III occupied the anchors of the ATP site, and only showed the competitive inhibitions for ATP. The pocket environment of ATP is generally conserved in kinase family, and the inhibitors of Group III also have the broadband inhibition for multiple kinases, including AG538 (**5**), which is observed on insulin-like growth factor-1 receptor (IGF-1R) [25], IR, EGFR [26], and Src kinases [27].

We sought to evaluate the binding-site features of the apo and closed forms that show a significant structural change because of LID closure and domain movement [19], [21], [28]. Indeed, the apo-form HpSK structure shows higher deviations in the LID region and in the adenine binding loop region (residues 143–150) (Fig. S3). Superimposition of the apo and closed HpSK structures revealed a tight binding pocket, particularly for the shikimate binding site. While the apo and closed forms had the same number of consensus anchors (E1, H1, H2, H3, V1 and V2), the spatial arrangements of these anchors were more packed in the closed form (Figs. 2C and 2D). Residues (D31 and D33) that contribute to H2 of the apo form were closer to each other in the closed conformation, resulting in a lower volume at this pocket. Likewise, the corresponding pocket at V2 surrounded by F48, G80, and G81 in HpSK had less space in the closed form, hindering the accommodation of large moieties in this pocket. The above evidences demonstrate that the induced LID conformation of shikimate kinases was sensitive in the structure-based drug discovery strategy (Fig. 4). The CoreSiMMap, considering both apo-form and close-form structures, can reduce the ill effects.

### Accuracy of the CoreSiMMap-based screening method

We next evaluated the accuracy of energy-based and CoreSiMMap-based scoring methods. The energy-based score (docking energy) of a compound was generated using a docking program, GEMDOCK [23], [29]. The scoring function in GEMDOCK is piecewise linear potential (PLP), which is a simple scoring function and is comparable to some energy-based scoring functions in estimating binding affinities [30]. Here, we used the hit rate and enrichment to assess the overall accuracy. The hit rate is defined as *A_h_*/*T_h_* (%) and the enrichment is (*A_h_*/*T_h_*)/(*A*/*T*), where *A_h_* is the number of active compounds among the *T_h_* highest ranking compounds (hit list), *A* is the total number of active compounds in the database, and *T* is the total number of compounds in the database. Here, A*_h_* is 8 (hits, compounds with IC_50_<100 µM) based on the bioassay results, and *T* is 6000 based on the 6000 top-ranked compounds from screening databases. We computed the average of enrichments, defined as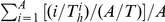, where 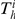 is the number of compounds in a hit list containing *i* active compounds.

**Figure 7:**
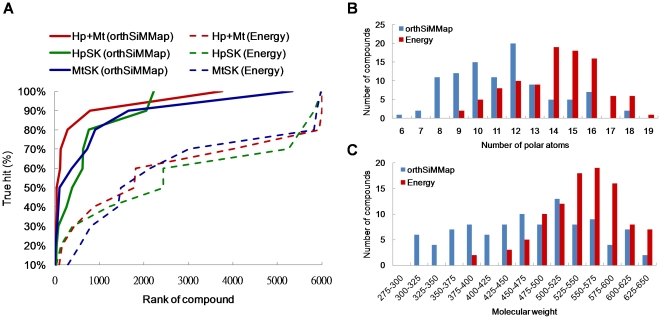
Performance of CoreSiMMap method on apo-form HpSK and MtSK. (A) True-hit rates of energy-based and CoreSiMMap scoring approaches. The CoreSiMMap scores (solid line) of adaptive inhibitors are significantly better than the energy-based scores (dashed line) for the 6000 top-ranked compounds by combining the Maybridge and NCI databases. (B) Distribution of number of polar atoms. (C) Molecular weights of top 100 compounds from CoreSiMMap scores and energy-based scores.

As shown in Figure 7A, the CoreSiMMap-based scoring method (solid lines) significantly outperformed the energy-based scoring method (dashed lines), which is often used in docking tools, when applied to apo-form HpSK and MtSK. The average enrichments of 3.73 (HpSK), 1.59 (MtSK), and 2.74 (fusion of HpSK and MtSK) were obtained using energy-based scoring method, as compared to 11.18 (HpSK), 35.51 (MtSK), and 93.69 (fusion of HpSK and MtSK), obtained using the CoreSiMMap scoring method. The average hit rates were 0.92% (HpSK), 0.21% (MtSK), and 0.37% (fusion of HpSK and MtSK) using energy-based scoring methods as compared to 34.13% (HpSK), 17.57% (MtSK), and 67.02% (fusion of HpSK and MtSK) using the CoreSiMMap score. Additionally, the CoreSiMMap-based RCS strategy was more accurate than the SiMMap-based strategy for a single target (HpSK or MtSK).

The CoreSiMMap scoring method can reduce the deleterious effects of screening ligand structures that are rich in charged or polar atoms. Generally, energy-based scoring functions favor the selection of high-molecular-weight compounds that yield high vdW potentials, as well as polar compounds that produce hydrogen-bonding and/or electrostatic potentials [23]. The average molecular weights of the 100 top-ranked compounds of the CoreSiMMap-based and energy-based scoring methods were 459.9 and 532.6, respectively; the average numbers of polar atoms were 11.3 (CoreSiMMap-based method) and 14.1 (energy-based method) (Figs. 7B and 7C). The ranks of the 10 active compounds were much higher in the CoreSiMMap-based scoring analysis than in the energy-based analysis. Notably, NSC162535 (**2**) was ranked as 1 and 1821 using the apo-form CoreSiMMap-based and energy-based scoring methods, respectively (Fig. 4).

## Discussion

By far the largest obstacle in structure-based drug discovery is the relatively low hit rate of scoring methods owing to the lack of adequate quantities of binding partners for a given target. The accuracy of a given individual scoring function is generally unknown and/or cannot be evaluated. The emphasis of the CoreSiMMap-based screening method developed here is therefore to provide a useful index to improve screening accuracy for the identification of adaptive inhibitors when the target proteins share conserved binding sites. Through the employment of this developed method, we successfully found six new potent inhibitors (<8.0 µM) of HpSK and MtSK. Two of the 65 kinase inhibitors were also found to inhibit both HpSK and MtSK activity. The finding that NSC45611 (**1**), NSC162535 (**2**), and NSC45612 (**3**) were competitive inhibitors of ATP and shikimate suggests that they belong to a novel class of shikimate kinase inhibitors. These results illustrate a robust CoreSiMMap-based screening approach for identifying selective kinase inhibitors.

The combined apo/closed CoreSiMMap analysis utilized here is considered to be useful for induced-fit P-loop kinases. Of six potent inhibitors, all except NSC45609 (**7**) have a higher rank in the apo-form than in the closed-form CoreSiMMap-based scoring analysis. Additionally, the top-ranked inhibitors according to the apo-form CoreSiMMap-based scoring analysis often have larger moieties (such as naphthalene or nitrobenzene) on both sides as opposed to those with a relatively small moiety (e.g., amide or aliphatic chain). The closed-form CoreSiMMap-based scoring analysis has, nonetheless, yielded useful hits, including NSC45609 (**7**) and SPB01099 (11). Target proteins with dynamic induced-fit forms, such as the P-loop SKs, represent a major limitation for the structure-based screening approach.

The CoreSiMMap that shares consensus binding environments among orthologous targets will be valuable for the development of effective common inhibitors. Indeed, the inhibitors identified herein had comparable IC_50_ values, docked poses, and competition properties for both HpSK and MtSK (Figs. 4, 5 and 6). More importantly, this CoreSiMMap, which includes both nucleotide and shikimate sites, specifically identified inhibitors that competed with both substrates, whereas the two general kinase inhibitors (AG538 (5) and GW5074 (12)) that did not compete well with the shikimate site. Notably, the potent competitive shikimate inhibitors have a common -N = N- moiety. A further lead optimization will be required to verify the importance of this moiety.

The developed CoreSiMMap-based screening method is database-independent. Comparable anchors were identified in compounds from the Maybridge and NCI databases. Each of the anchors also included similar chemical moieties. Nonetheless, the derived proportion of these moieties was different because the Maybridge and NCI databases contain heterogeneous distribution of compounds. For example, the proportions of carboxyl, sulfonate, and phosphate were significantly higher in compounds from the NCI database than in those from the Maybridge database. On the other hand, the derived model was sensitive to binding-site properties, as revealed by the difference between the apo- and closed-form models (Fig. 2). In summary, we anticipate that the CoreSiMMap-based screening method will be useful for discovering new inhibitors, investigating binding mechanisms, and guiding lead optimization for orthologous targets.

### Conclusions

We have developed a CoreSiMMap-based screening method to derive conserved pocket-moiety environments (e.g., hot spots) between orthologous targets and inhibitors from screening compound libraries. Studies of site-directed mutagenesis and analogues revealed that critical conserved interacting residues of pocket-moiety interaction anchors participate in biological functions. Experimental results reveal that the CoreSiMMap-based screening method is database-independent and the CoreSiMMap scoring method significantly outperformed the energy-based scoring method, which is often used in docking tools. Furthermore, we have successfully obtained several potent inhibitors and revealed the binding mechanisms for MtSK and HpSK. The CoreSiMMap is useful in providing biological insights and guiding the process of drug discovery, including hit search and lead optimization.

## Materials and Methods

### Overview of CoreSiMMap-based screening method

Figure 1 shows the main steps of the CoreSiMMap-based screening method. For each orthologous target, we first docked selected compounds into the binding site using GEMDOCK, which is an in-house molecular docking program that uses PLP to measure intermolecular potential energy between proteins and compounds [29]. Our previous works showed that GEMDOCK yields very comparable results to some other docking tools (e.g., DOCK, FlexX, and GOLD) when applied to 100 protein-ligand complexes and some virtual screening targets [29], [30]. Additionally, GEMDOCK has been successfully applied to identify novel inhibitors and binding sites for some targets [31]–[34]. After the docking procedure, we used the top 2% (∼6,000) of compounds based on docking energy to analyze target-compound interaction profiles (Fig. 1B and Fig. 3) to establish the site-moiety map (Figs. 2A and 2B). Each anchor represented a local binding environment with a specific physico-chemical property or pharmacophore spot, which was derived by identifying statistically significant interacting residues and compound moieties. The CoreSiMMap (Figs. 2C and 2D) that consists of the aligned anchors of orthologous proteins is generated by extracting the consensus anchors from the orthologous SiMMaps.

### Preparations of SK structures of four target proteins and screening databases

Four structures of the target proteins were selected from Protein Data Bank (PDB) for virtual screening. They were the apo-form structure (PDB code: 1ZUH[19]) and the closed-form structure (PDB code: 1ZUI[19]) of HpSK, and the apo-form structure (PDB code: 2IYT[21]) and the closed-form structure (PDB code: 1ZYU[24]) of MtSK. The residues of the binding sites of these four structures were prepared by the following steps. Firstly, the apo-form and closed-form structures of MtSK were aligned to the closed-form structure of HpSK using the structural alignment tool [35]. The bound ligands (SKM and ACP) of HpSK were used to determine the binding sites of these four structures. For each structure, the residues of the binding site were obtained by considering the protein atoms that were ≤10 Å from these two bound ligands.

We selected compounds from the Maybridge and NCI databases for both HpSK and MtSK to establish the site-moiety maps and to identify novel candidates. In total, 65,947 (Maybridge) and 236,962 (NCI) compounds with molecular weights between 200 and 650 dalton were prepared for screening. In addition, we collected a dataset of 37 orthologous target pairs with biological function annotations (*e.g.*, substrate binding, metal binding, and catalytic residues) summarized from UniProt [36], Consurf server [37], and Catalytic Site Atlas [38] (Table. S1) to verify the consensus anchors residues that were derived using the CoreSiMMap-based screening method. Experimental results reveal that the consensus anchors often represent the conserved binding environments that are involved in biological functions, such as substrate binding, metal binding, or catalytic functions. Residues of consensus anchors of these 37 orthologous pairs are often the key residues (*i.e.*, substrate binding residues, metal binding residues, catalytic residues, or high conserved residues with Consurf conservation score 8 or 9) or key anchors (*i.e.*, anchors that contain key residues) (Fig. S4). For example, D81, D84, and D201 of inositol-1-monophosphatase of *Methanocaldococcus jannaschii* (D82, D85, and D200 in *Archaeoglobus fulgidus*) are the consensus anchor residues and coordinate two metal ions for catalysis [39]. Another example is the androgen receptor of *Homo sapiens*, which is a target in the treatment of prostate cancer [40]. Three residues (N705, R752, and T877) of the consensus anchors are essential for the ligand binding [41]. These results reveal that the consensus anchor residues often play important roles in biological functions.

### CoreSiMMap-based screening method

The main steps of the CoreSiMMap-based method for producing SiMMaps and a CoreSiMMap from orthologous targets are described as follows (Fig. 1):

- Virtual screening of orthologous targets. We used in-house GEMDOCK program [23], [29] to screen Maybridge (65,947 compounds) and NCI (236,962 compounds) databases for four targets, including apo and closed forms of HpSK and MtSK. GEMDOCK first assigned the formal charge and atom type (*i.e.,* donor, acceptor, both, or nonpolar) based on physico-chemical property of each atom of both compounds and binding sites. GEMDOCK then utilizes PLP to measure the intermolecular potential energies between the binding sites (rigid) and the compounds (flexible). Finally, these docked compounds were ranked in order of energy. The top 2% (∼6,000) of compounds of each target were selected from the screening results for subsequent protein-compound profiling. A personal computer cluster (80 nodes, each with an Intel Woodcrest 2.66 GHz processor and 4 GB of RAM) was used to implement the docking procedure. On average, a docking run (in which a compound was docked into a binding site of a protein) took 63 s; therefore, docking 302,909 compounds into these four proteins took about 11 days (∼1,200,000 docking runs).
- Selection of a diverse set of top-ranked compounds. To maintain a wide range of potential functional groups in the sampling of anchors, a cluster method was utilized to cluster the top-ranked compounds. A hierarchical clustering method that exploited the topological features of the compounds was used. The topological features were generated by the atom pair (AP) approach [42], [43]. An AP was a substructure of a compound and was presented as “atom type I – bond distance – atom type J”, where atom types I and J are the atom types of atoms I and J, respectively, and bond distance is the number of bonds measured along the shortest path between atoms I and J. Atoms were classified into 10 types based on their chemical properties (Table. S2), and there are 55 different combinations of these 10 atom types in total. The value of the AP was set to 1 (ON) if the compound contained the substructure; otherwise, the value was set to 0. Here, the maximum number of the bonds was set to 15. Therefore, the number of APs was 825 (55×15), and the topology of a compound was represented as a string of 825 binary bits.

Subsequently, the AP binary strings of the top-ranked compounds were used to cluster the compounds in a hierarchical clustering procedure[44]. In the procedure, we firstly used the Tanimoto coefficient (*Tc*) [43] to quantify the similarity between two compounds (A and B). *Tc* was defined as
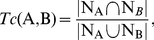
(1)where |N_A_∩N_B_| is the number of ON bits that are common to both A and B, and | N_A_ ∪ N_B_ | is the number of ON bits in either A or B. Based on the Equation (1), the similarity matrix of the compounds was generated, and applied to construct a dendrogram by using complete linkage hierarchical clustering. The *Tc* threshold that was used to group compounds was set to 0.9. The compound with the lowest energy in each group was selected as the representative compound for the group. Accordingly, we could select a diverse set of compounds to ensure that many functional groups would form the anchors.

- Profiling analysis of target-compound interactions (Fig. 1B and Fig. 3). The profiles described the interactions (*i.e.*, electrostatic (E), hydrogen-bonding (H), and van der Waals (V) interactions) between the diverse top-ranked compounds and the protein residues, from which the anchors could be derived. The target-compound interactions were identified by applying the PLP of GEMDOCK program [29]. In the E or H profiles, a profile entry was set to 1 if the compound forms electrostatic or hydrogen-bonding interactions with the residue (green regions); otherwise, the entry was set to 0 (black regions). I the V profile, a profile entry was set 1 if the V energy was less than −4 kcal/mol.
- Identification of anchors (Fig. 1C). We identified consensus interactions between residues and compound moieties from the profiles. For an interacting residue, we used the Z-score to measure the interacting conservation between the residue and moieties. The interacting conservation is treated as a binomial distribution, which is approximated as a normal distribution when either *p*≤0.5 and *np*>5 or *p*>0.5 and *n*(1−*p*)>5, where *n* is the number of selected compounds and *p* is the probability of forming an interaction. Theoretically, at least 500 compounds should be selected to construct a target-compound interaction profile. Spatially neighboring interacting residues and moieties with statistically significant Z-score≥1.645 are referred as an anchor. A set of anchors derived from the target-compound interaction profile can be used to establish a site-moiety map for each orthologous target.
- Establishment of CoreSiMMap of orthologous targets (Figs. 1D, 1E and Fig. 2). Firstly, the apo-form and closed-form structures of orthologous proteins (HpSK and MtSK) were aligned using a structural alignment tool [35]. The superimposed SiMMaps (anchors) of the orthologous proteins revealed an overlapping region of matched anchors which form the CoreSiMMap (Figs. 2C and 2D). For compound *x*, the CoreSiMMap-based score, combining anchor (CoreSiMMap) and PLP (GEMDOCK) scores, is defined as 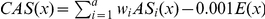, where *w_i_* is the conservation of the anchor *i* on orthologous targets; *AS_i_*(*x*) is the anchor score of compound *x* in anchor *i*; *a* is the number of anchors, and *E*(*x*) is the docked energy of the compound *x*. The CoreSiMMap-based rank of compound *x* was obtained by arranging the compounds in order of descending *CAS*(*x*). Table S3 presents the parameters that are used to construct the CoreSiMMap.
- Inhibition assay. We selected top-ranked compounds using rank-based consensus scoring (RCS) for subsequent inhibitory assay. The RCS of a compound *x* was calculated by combining the ranks of *m* (apo/closed) forms of *n* orthologous targets as follows: 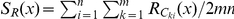, where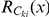 is the CoreSiMMap-based rank of the compound *x* on the *k* (apo/closed) form of target *i*. Here, *m* and *n* are 2. We obtained the RCS rank of the compound *x* by arranging the compounds in order of ascending *S_R_*(*x*).
- Refinement of CoreSiMMaps. Active and inactive compounds from the enzyme inhibition assay were used to evaluate and refine the CoreSiMMaps.

### Cloning, expression, and purification of *M. tuberculosis* shikimate kinase (MtSK) and *H. pylori* shikimate kinase (HpSK)

*AroK*, which encodes MtSK, was amplified from the chromosomal DNA of *Mycobacterium tuberculosis* str. Haarlem with PCR using pfu DNA polymerase. Primers (MtSK-F (forward), 5′-CGCGGATCCATGGCACCCAAAGCGGTTCTCGTCG-3′; MtSK-R (reverse), 5′-AAACTGCAGTCATGTGGCCGCCTCGCTGGGGCTG-3′) that contain sequences for the *Bam*HI and *Pst*I sites, respectively, were designed based on the nucleotide sequence of the reported MtSK gene of *M. tuberculosis* (accession number AASN01000059; Genome Project: 17353). The amplified fragment was inserted into the pET28a expression vector to generate pET28a-MtSK containing an N-terminal six-histidine tag (His_6_) for purification purposes. The recombinant MtSK in *E. coli* BL-21 (DE3) cells was induced with 1 mM isopropyl-β-D-thiogalactopyranoside (IPTG) at 16°C. Bacterial pellets were fractionated, and soluble proteins in cytosolic fractions were collected. The expressed MtSK protein with a His_6_ tag was purified by immobilized-nickel ion chromatography, followed by Superdex-75 gel filtration chromatography (Pharmacia), and then analyzed using SDS-PAGE to verify its purity. The cloning and purification of HpSK were based on previous methods [19].

### Preparation of mutant HpSKs

Site-directed mutagenesis was performed using the overlap extension PCR [45] method with the plasmid pQE30-HpSK as the template. All the mutations were confirmed by sequencing of the whole ligated PCR fragment. Mutant proteins were expressed and purified by the same procedures as described for the wild-type HpSK.

### Enzymatic activity assay and analysis of inhibitor kinetics

Shikimate kinase activity was determined by coupling the release of ADP from the SK-catalyzed reaction to the oxidation of NADH using pyruvate kinase (EC 2.7.1.40) and lactate dehydrogenase (EC 1.1.1.27) as coupling enzymes [46]. Shikimate-dependent oxidation of NADH was measured by monitoring the decrease in *A*340 (ε = 6,200 M^−1^ cm^−1^). The assay was carried out at 25°C in a mixture of 100 mM Tris-HCl buffer, pH 7.5, 50 mM KCl, 5 mM MgCl_2_, 1.6 mM shikimic acid, 2.5 mM ATP, 1 mM phosphoenolpyruvate (PEP), 0.1 mM NADH, 2.5 units of pyruvate kinase per ml, and 2.7 units of lactate dehydrogenase per ml. All assays were conducted in a 96-well microplate and analyzed with a spectrophotometer (FLUOstar OPTIMA, BMG LABTECH).

Stock solutions of compounds were prepared in dimethylsulfoxide (DMSO). Each set of measurements included 10% DMSO as a negative control. Approximately 80 nM enzyme was added to a reaction of 200 µl containing compounds. The initial velocities of enzyme activity were determined in the presence of various concentrations of test compounds to investigate the dose-dependent inhibition effects. The IC_50_ values of these compounds were obtained by fitting the data to a sigmoid dose-response equation in GraphPad Prism 4.

After the preliminary screening, the inhibitor modality was determined by measuring the effects of inhibitor concentrations on the enzymatic activity as a function of substrate concentration. In the inhibition experiment in which the ATP concentration was fixed at 2.5 mM, shikimate was a varied substrate (0.06, 0.12, 0.24, 0.48, and 0.96 mM) when the concentration of inhibitor was varied from 0 to 50 µM. In parallel, in the inhibition experiment in which the shikimate concentration was fixed at 1.6 mM, ATP was a varied substrate (0.06, 0.12, 0.24, 0.48, and 0.96 mM) when the concentration of inhibitor was varied from 0 to 50 µM. A nonlinear least square-fitting algorithm was applied to the absorbance data to determine the kinetic mechanism data.

## Supporting Information

Figure S1 **Site-moiety maps of apo-form (A) HpSK and (B) MtSK.** Each anchor represents one of three binding environments (electrostatic: blue; hydrogen-bonding: green; van der Waals: black). The distribution of identified chemical moieties for each anchor is shown as a pie chart. In HpSK, H1, V1, and H2 are situated at the nucleotide site, while H3, V2, and E1 are at the shikimate site. In MtSK, H1, V1, V3, and H2 are at the nucleotide site, while H3, V2, and E1 are at the shikimate site.
(TIF)

Figure S2 **Site-moiety maps of closed-form (A) HpSK and (B) MtSK.** Each anchor represents one of three binding environments (electrostatic: blue; hydrogen-bonding: green; van der Waals: black). The distribution of identified chemical moieties for each anchor is shown as a pie chart. In HpSK, H1, V1, and H2 are situated at the nucleotide site, while H3, V2, and E1 are at the shikimate site. In MtSK, H1, V1, V3, and H2 are at the nucleotide site, while H3, V2, and E1 are at the shikimate site.
(TIF)

Figure S3 (A) Circular dichroism profiles of HpSK in the presence or absence of various ligands. (B) Superimposition of apo and closed HpSK structures. Apo and closed structures are shown in red and green, respectively. Shikimate and phosphate are represented as sticks. The carbon, oxygen and phosphorus atoms are colored green, red, and orange, respectively. Pharmacophore spots of the apo (C) and closed (D) forms of HpSK.
(TIF)

Figure S4 (A) The percentages of key residues of consensus anchor residues and non-consensus anchor residues derived from the 37 orthologous target pairs. Key residues are substrate binding residues, metal binding residues, catalytic residues, or high conserved residues. (B) The percentages of key anchors of consensus anchors and non-consensus anchors derived from the 37 orthologous target pairs. Key anchors are anchors that contain one or more key residues.
(TIF)

Table S1 **Summary of 37 pairs of orthologous targets.**
(DOC)

Table S2 **Atom types used for atom pair descriptors.**
(DOC)

Table S3 **Parameters used in the CoreSiMMap.**
(DOC)
