# Supplementary figures and images for: Core Site-Moiety Maps Reveal Inhibitors and Binding Mechanisms of Orthologous Proteins by Screening Compound Libraries
Source: PLoS One. 2012 Feb 29;7(2):e32142. doi: 10.1371/journal.pone.0032142 (PMC3290551; doi:10.1371/journal.pone.0032142)

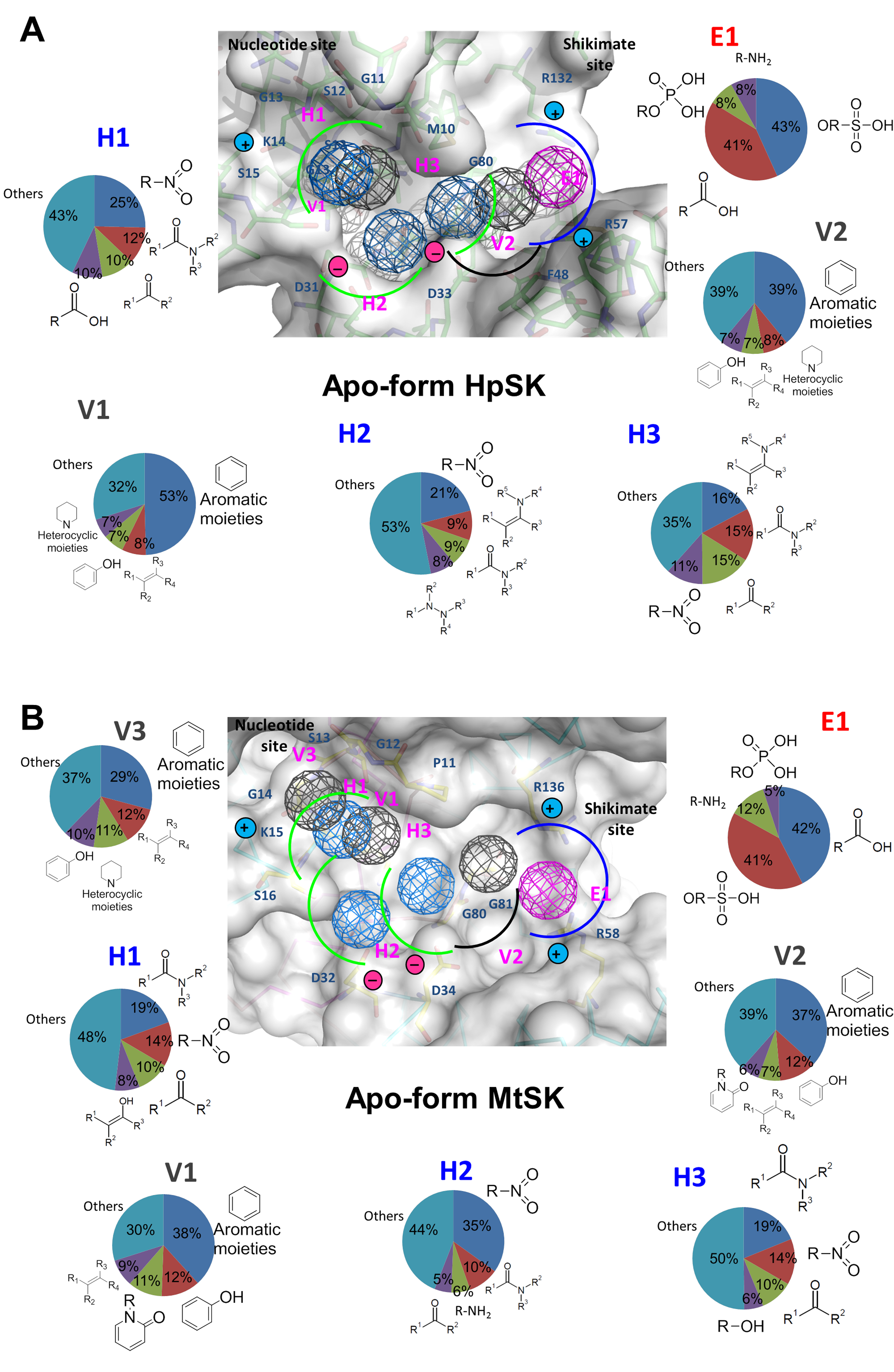

Supplement: Figure S1 — Site-moiety maps of apo-form (A) HpSK and (B) MtSK. Each anchor represents one of three binding environments (electrostatic: blue; hydrogen-bonding: green; van der Waals: black). The distribution of identified chemical moieties for each anchor is shown as a pie chart. In HpSK, H1, V1, and H2 are situated at the nucleotide site, while H3, V2, and E1 are at the shikimate site. In MtSK, H1, V1, V3, and H2 are at the nucleotide site, while H3, V2, and E1 are at the shikimate site. (TIF) [file pone.0032142.s001.tif]

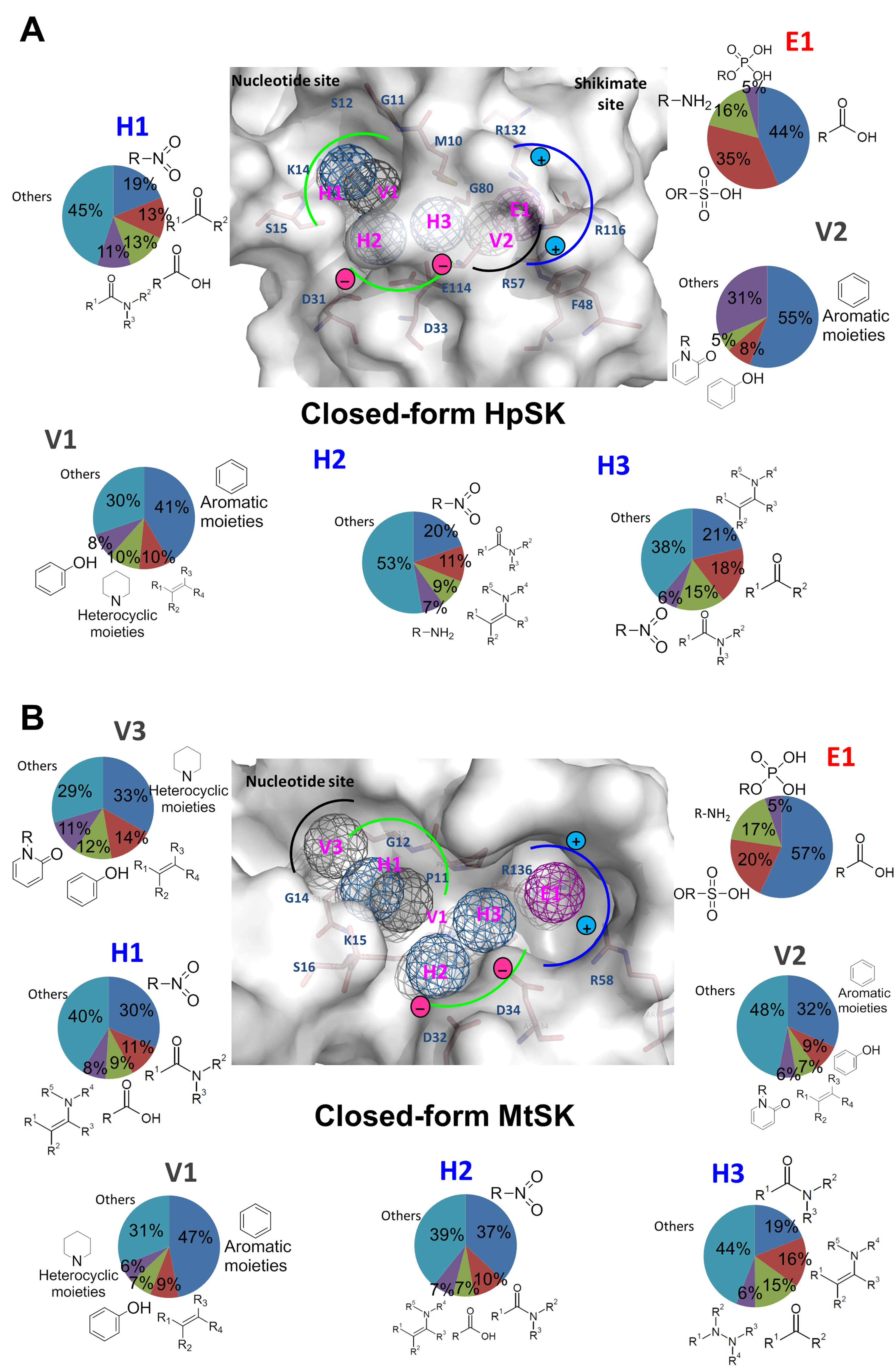

Supplement: Figure S2 — Site-moiety maps of closed-form (A) HpSK and (B) MtSK. Each anchor represents one of three binding environments (electrostatic: blue; hydrogen-bonding: green; van der Waals: black). The distribution of identified chemical moieties for each anchor is shown as a pie chart. In HpSK, H1, V1, and H2 are situated at the nucleotide site, while H3, V2, and E1 are at the shikimate site. In MtSK, H1, V1, V3, and H2 are at the nucleotide site, while H3, V2, and E1 are at the shikimate site. (TIF) [file pone.0032142.s002.tif]

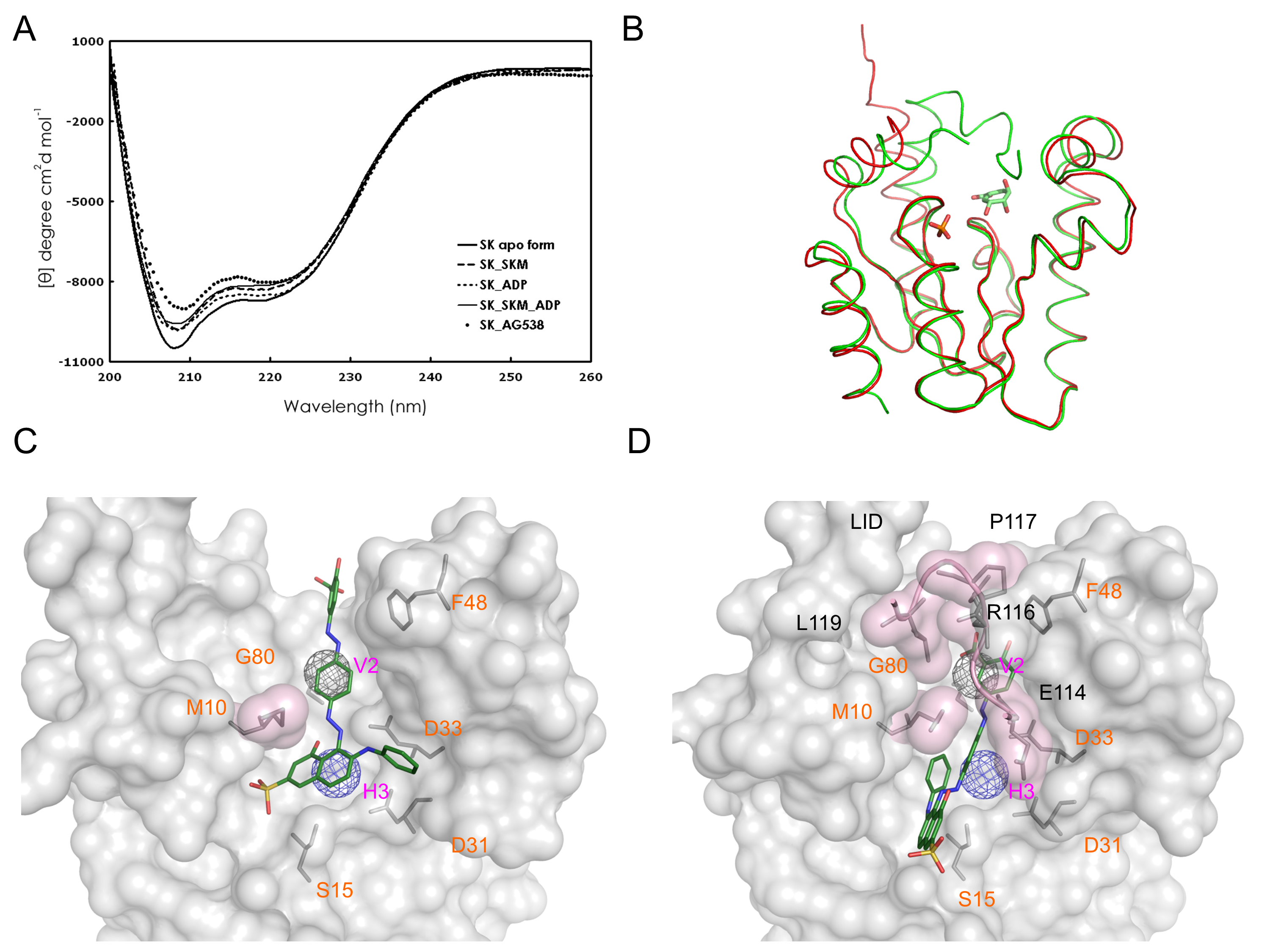

Supplement: Figure S3 — (A) Circular dichroism profiles of HpSK in the presence or absence of various ligands. (B) Superimposition of apo and closed HpSK structures. Apo and closed structures are shown in red and green, respectively. Shikimate and phosphate are represented as sticks. The carbon, oxygen and phosphorus atoms are colored green, red, and orange, respectively. Pharmacophore spots of the apo (C) and closed (D) forms of HpSK. (TIF) [file pone.0032142.s003.tif]

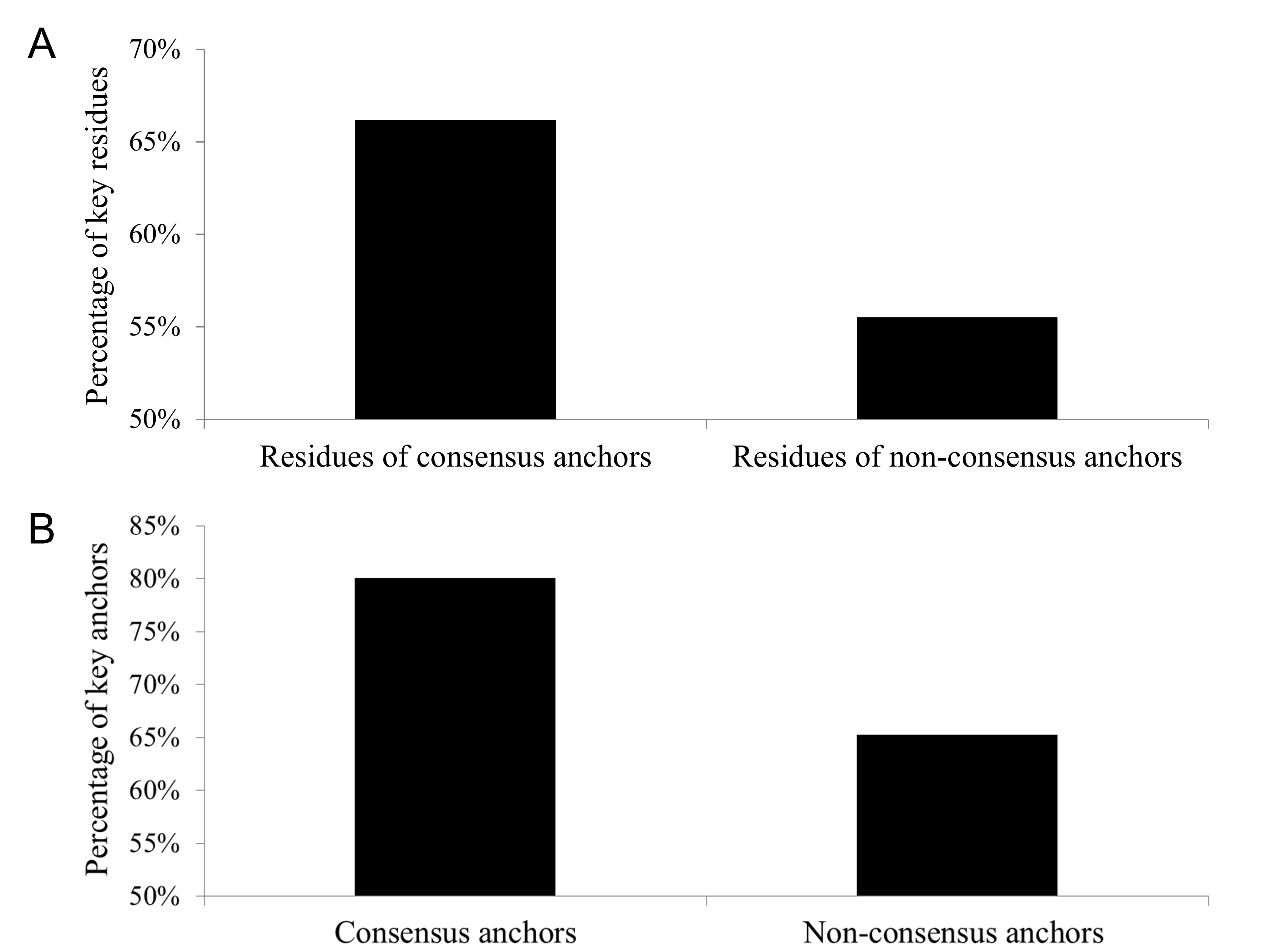

Supplement: Figure S4 — (A) The percentages of key residues of consensus anchor residues and non-consensus anchor residues derived from the 37 orthologous target pairs. Key residues are substrate binding residues, metal binding residues, catalytic residues, or high conserved residues. (B) The percentages of key anchors of consensus anchors and non-consensus anchors derived from the 37 orthologous target pairs. Key anchors are anchors that contain one or more key residues. (TIF) [file pone.0032142.s004.tif]
