# Supplementary material for: Core Site-Moiety Maps Reveal Inhibitors and Binding Mechanisms of Orthologous Proteins by Screening Compound Libraries
Source: PLoS One. 2012 Feb 29;7(2):e32142. doi: 10.1371/journal.pone.0032142 (PMC3290551; doi:10.1371/journal.pone.0032142)
Supplement: Table S1 — Summary of 37 pairs of orthologous targets. (DOC) [file pone.0032142.s005.doc]

Table S1. Summary of 37 pairs of orthologous targets

| ID | Description | Gene Name | Species | | PDB code | | Bound ligand |
| --- | --- | --- | --- | --- | --- | --- | --- |
|  |  |  | A | B | A | B |  |
| 1 | 3-phosphoshikimate 1-carboxyvinyltransferase | aroA | Escherichia coli | Agrobacterium sp. | 1x8t | 2pqc | RC1 |
| 2 | Adenosine deaminase | Ada | Mus musculus | Bos taurus | 1a4m | 1krm | PRH |
| 3 | Androgen receptor | AR | Homo sapiens | Rattus norvegicus | 1t5z | 1i37 | DHT |
| 4 | Arginase-1 | ARG1 | Homo sapiens | Rattus norvegicus | 2pll | 1d3v | ABH |
| 5 | Aspartate aminotransferase | aspC | Thermus thermophilus | Escherichia coli | 1bkg | 1aia | PMP |
| 6 | ATP-dependent hsl protease ATP-binding subunit hslU | hslU | Escherichia coli | Haemophilus influenzae | 1do0 | 1g3i | ATP |
| 7 | Bifunctional protein glmU | glmU | Haemophilus influenzae | Escherichia coli | 2v0i | 1fwy | UD1 |
| 8 | cAMP-dependent protein kinase catalytic subunit α | Prkaca | Mus musculus | Bos taurus | 1atp | 1q24 | ATP |
| 9 | Cytochrome b | MT-CYB | Gallus gallus | Bos taurus | 3l71 | 1sqb | AZO |
| 10 | Dihydrofolate reductase | DHFR | Homo sapiens | Mus musculus | 3gyf | 3k47 | D09 |
| 11 | Dihydrofolate reductase | folA | Escherichia coli | Mycobacterium tuberculosis | 1ddr | 1df7 | MTX |
| 12 | DNA mismatch repair protein mutS | mutS | Escherichia coli | Thermus aquaticus | 1e3m | 1fw6 | ADP |
| 13 | Elongation factor Tu-A | tufA | Thermus thermophilus | Escherichia coli | 1ha3 | 1d8t | GDP |
| 14 | Fatty acid-binding protein, adipocyte | Fabp4 | Mus musculus | Homo sapiens | 1lie | 2hnx | PLM |
| 15 | Fructose-1,6-bisphosphatase 1 | FBP1 | Sus scrofa | Homo sapiens | 1eyj | 1fta | AMP |
| 16 | Glucose-6-phosphate isomerase | Gpi | Mus musculus | Oryctolagus cuniculus | 2cxr | 1dqr | 6PG |
| 17 | Hemagglutinin-neuraminidase | HN | Newcastle disease virus | Newcastle disease virus | 1e8v | 1usr | DAN |
| 18 | HTH-type transcriptional regulator qacR | qacR | Staphylococcus aureus | Staphylococcus aureus | 1jt6 | 3br1 | DEQ |
| 19 | Inositol-1-monophosphatase | suhB | Methanocaldococcus jannaschii | Archaeoglobus fulgidus | 1g0h | 1lbx | IPD |
| 20 | Methionine aminopeptidase | map | Escherichia coli | Mycobacterium tuberculosis | 1xnz | 3iu7 | FCD |
| 21 | Neuraminidase | NA | Influenza A virus (H11N9) | Influenza A virus (H1N1) | 1nnc | 3b7e | ZMR |
| 22 | NH(3)-dependent NAD(+) synthetase | nadE | Bacillus subtilis | Bacillus anthracis | 1ih8 | 2pz8 | APC |
| 23 | Orotidine 5'-phosphate decarboxylase | pyrF | Methanobacterium thermoautotrophicum | Pyrococcus horikoshii | 1lol | 2czf | XMP |
| 24 | Peptide deformylase | def | Escherichia coli | Leptospira interrogans | 1g2a | 1szz | BB2 |
| 25 | Protein farnesyltransferase subunit beta | Fntb | Rattus norvegicus | Homo sapiens | 1d8d | 1tn6 | FII |
| 26 | Protein recA | recA | Mycobacterium smegmatis | Mycobacterium tuberculosis | 1ubg | 1mo6 | DTP |
| 27 | Purine nucleoside phosphorylase | PNP | Bos taurus | Homo sapiens | 1a9s | 1rct | NOS |
| 28 | Pyridoxal kinase | PDXK | Homo sapiens | Ovis aries | 2yxu | 1lhr | ATP |
| 29 | Ribulose bisphosphate carboxylase large chain | rbcL | Nicotiana tabacum | Spinacia oleracea | 1rlc | 1ir1 | CAP |
| 30 | Shikimate kinase | aroK | Helicobacter pylori | Mycobacterium tuberculosis | 1zui | 1zyu | SKM/  S3P |
| 31 | Thymidine kinase | TK | Human herpesvirus 1 | Equine herpesvirus 4 | 1e2j | 1p6x | THM |
| 32 | Thymidylate synthase | thyA | Escherichia coli | Lactobacillus casei | 1aiq | 1lca | CB3 |
| 33 | Tyrosine-protein kinase ABL1 | Abl1 | Mus musculus | Homo sapiens | 1opk | 1opl | P16 |
| 34 | UDP-glucose 4-epimerase | galE | Escherichia coli | Homo sapiens | 1lrj | 1hzj | UD1 |
| 35 | UDP-N-acetylglucosamine 1-carboxyvinyltransferase | murA | Enterobacter cloacae | Escherichia coli | 1ryw | 3iss | EPU |
| 36 | Vitamin D3 receptor | VDR | Homo sapiens | Rattus norvegicus | 1db1 | 1rk3 | VDX |
| 37 | Xylose isomerase | xylA | Streptomyces rubiginosus | Arthrobacter sp. | 1xig | 1xlc | XYL |
