# Supplementary material for: Core Site-Moiety Maps Reveal Inhibitors and Binding Mechanisms of Orthologous Proteins by Screening Compound Libraries
Source: PLoS One. 2012 Feb 29;7(2):e32142. doi: 10.1371/journal.pone.0032142 (PMC3290551; doi:10.1371/journal.pone.0032142)
Supplement: Table S2 — Atom types used for atom pair descriptors. (DOC) [file pone.0032142.s006.doc]

Table S2. Atom types used for atom pair descriptors

| Atom type | Description |
| --- | --- |
| C.ar | Carbons on an aromatic ring |
| C.nar | Carbons on an nonaromatic ring |
| N.ar | Nitrogens on an aromatic ring |
| N.nar | Nitrogens on an aromatic ring |
| O.3 | Sp3 hybridized oxygens |
| O.2 | Sp2 hybridized oxygens |
| S | Sulfur atoms |
| P.3 | Phosphorus atoms |
| X | Halogen atoms |
| Oa | Other atoms |
