# Supplementary material for: Core Site-Moiety Maps Reveal Inhibitors and Binding Mechanisms of Orthologous Proteins by Screening Compound Libraries
Source: PLoS One. 2012 Feb 29;7(2):e32142. doi: 10.1371/journal.pone.0032142 (PMC3290551; doi:10.1371/journal.pone.0032142)
Supplement: Table S3 — Parameters used in the CoreSiMMap. (DOC) [file pone.0032142.s007.doc]

Table S3. Parameters used in the CoreSiMMap

| Item | Parameters and Description |
| --- | --- |
| Hydrogen-bonding interactions | An interaction entry of the H profile was set to 1 if a residue formed a hydrogen-bonding interaction (s) with a compound; conversely; the entry was set to 0. The interactions were determined by GEMDOCK. |
| Electrostatic interactions | An interaction entry of the profile was set to 1 if a residue formed an electrostatic interaction (s) with a compound; conversely; the entry was set to 0. The interactions were determined by GEMDOCK. |
| van der Waals interactions | An interaction entry of the profile was set to 1 if the interaction energy between a residue and a compound is less than -4 (kcal/mol). The interaction energy was generated by GEMDOCK. In GEMDOCK, the energies of stacking ring-ring interactions or ring-*n* carbon chain interactions (*n* ≥ 3) are less than -4 (kcal/mol). |
| Z-score threshold | Z-score threshold is set to 1.645 (95% confidence level) using Student's *t*-test |
